# Credit-frequency compliance paradox and dual-track hazard evolution in urban occupational health

**DOI:** 10.3389/fpubh.2025.1688643

**Published:** 2025-12-19

**Authors:** Xiaoting Zhu, Junjie Ye, Shihong Sun, Chunming Liu, Wenfeng Cai

**Affiliations:** Guangzhou Tianhe District Center for Disease Control and Prevention (Guangzhou Tianhe District Health Supervision Institute), Guangzhou, Guangdong, China

**Keywords:** credit-frequency compliance paradox, dual-trackhazard evolution, hybrid governance mechanisms, noise exposure, occupational health hazards, post-industrial cities

## Abstract

**Purpose:**

This study investigates occupational health compliance in post-industrial urban areas, focusing on noise hazards within Guangzhou’s Tianhe District (2020–2024). We propose two novel concepts: the Credit-Frequency Compliance Paradox, where small enterprises exhibit unexpectedly higher hazard-monitoring compliance compared to larger firms under hybrid governance; and Dual-Track Hazard Evolution, highlighting simultaneous declines in conventional occupational hazards (dust) and rises in emerging risks (noise and unique chemical exposures).

**Methods:**

Using data from the “Guangdong Province Occupational Health Quality Control Platform,” we analyzed monitoring coverage rates, enterprise exceedance rates, and hazard exceedance rates, stratified by enterprise size and industry.

**Results:**

Small and micro enterprises showed higher periodic monitoring coverage (47.35–50.60%) than large and medium-sized firms (22.22–24.18%) (χ^2^_trend = 16.987, *p* < 0.001), validating the Compliance Paradox. Although overall hazard exceedance rates significantly decreased annually (χ^2^_trend = 4.965–10.386, *p* < 0.05), critical subsector hazards persisted: 75.00% silica dust in clay brick and block manufacture and 12.50% chemical exceedances in unclassified service industries. Noise was the predominant physical hazard, impacting large enterprises (12.80% exceedance) and scientific research and technical services sectors [median: 81.60 dB(A)], where exceedance rates surged from 2.04 to 37.50% (χ^2^_trend = 14.318, *p* < 0.001) and noise intensity markedly increased (Jonckheere-Terpstra = 2532.000, *p* < 0.001) from 2020–2024. Significant variation existed across enterprise sizes (H = 55.140, *p* < 0.001) and industries (H = 254.964, *p* < 0.001).

**Conclusion:**

This study provides empirical support for the Credit-Frequency Compliance Paradox and documents Dual-Track Hazard Evolution—traditional occupational hazards decline while novel risks emerge in high-tech and service sectors. Although these frameworks originate from a single region, they may still offer preliminary insights into occupational health governance in other post-industrial urban contexts before being validated in diverse geographical and economic settings.

## 1 Introduction

Occupational diseases remain a serious global burden. The International Labor Organization (ILO) estimated that approximately 2.93 million deaths each year are attributable to occupational hazards, underscoring the critical importance of effective workplace health governance (1). The health of workers in China has historically been jeopardized by workplace hazards such as dust, chemical pollutants, and noise, especially in rapidly industrializing regions like Guangdong Province (2). In Guangdong Province, the predominant occupational affliction is noise-induced hearing loss, with a rising incidence of new cases annually (3, 4). Rapid industrialization—coupled with a boom in high-tech research and service sectors—contributes to a complex noise risk landscape (5). This is particularly evident in Guangzhou’s Tianhe District, a post-industrial hub characterized by integrating manufacturing, high-tech business, and services. This district generated a GDP of 655 billion yuan (approximately US$91 billion), ranking first among all districts in Guangzhou for the 17th consecutive year (6). The tertiary sector accounted for over 93% of GDP, while manufacturing contributed less than 7%, indicating a service-dominated economic structure (7). The district hosts more than 600,000 firms, representing 26% of Guangzhou’s total (6). Notably, 98.1% of occupational noise-induced hearing loss cases in Guangdong Province are linked to manufacturing, with large and medium-sized enterprises comprising 65.3% of these occurrences (4). However, emerging risks in scientific research and technical services sectors —often overshadowed by traditional industries—demand urgent attention.

Regular occupational hazard monitoring has emerged as a fundamental tool for the prevention and control of occupational diseases (8). By systematic assessment of dust, chemical, and physical exposures, such monitoring allows timely implementation of preventive and corrective measures—minimizing the risk of occupational diseases (8). However, coverage remains critically low. A 2022 study indicates that the coverage of periodic monitoring in Guangdong Province was only at 5.9% (2). Even Guangzhou, one of the top three cities in the province by enterprise count, achieved only 4.4% coverage (2). This reflects that Guangzhou’s occupational health regulation framework has failed to adapt to the swiftly changing requirements of industrial advancement and contemporary administration (8). Moreover, despite national legislation requiring routine hazard assessments, varying levels of compliance persist across enterprise sizes, raising concerns about the effectiveness of local governance strategies (2, 8).

Previous studies have consistently demonstrated that small enterprises generally have reduced adherence to occupational health rules in comparison to larger firms. Studies from different regions reflects this pattern: Liu et al. (9) found that among 5,328 enterprises in Kunshan city, larger firms had markedly greater monitoring rates compared to smaller ones. Similarly, Hu et al. (10) observed this same compliance gradient in Dongguan city, where large and medium firms exhibited 100% compliance, in contrast to 91.50 and 66.67% compliance rates in small and micro enterprises, respectively. This trend transcends Asia, with global research confirming that small enterprises often prioritize organizational survival over health and safety due to competing administrative priorities and constrained resources (9). Numerous small-company owners regard occupational safety regulations as primarily the employee’s responsibility and consider compliance requirements as financial burdens (11). The European Agency for Safety and Health at Work (2018) has reported similar observations in Europe, attributing these issues to insufficient regulatory awareness and the absence of formalized approaches among smaller firms (12). The U.S. Department of Justice likewise pointed out that small enterprises generally carry out less frequent and less formalized hazard monitoring than larger firms (13). This results from a shortage of specialist staff and frequent dependence on informal procedures in small firms, whereas large organizations allocate more formal operations and upgraded resources (13). Empirical research further supports that small enterprises possess less motivations and capabilities for compliance, partly due to reduced monitoring from regulators and a decreased likelihood of facing enforcement compared to larger firms (14).

Beyond these traditional patterns, however, emerging evidence suggests a more complex reality. The “Credit-Frequency Compliance Paradox” challenges established compliance theories as documented by recent global research. Recent systematic reviews indicate that small and medium-sized enterprises (SMEs) react distinctively to regulatory systems compared to larger firms, generally achieving superior compliance under targeted interventions (15–17). This contradictory pattern arises specifically within hybrid governance systems that integrate credit-based penalties with high-frequency monitoring, resulting in an inversion of traditional size-compliance relationships (17, 18). Specifically, hybrid governance refers to regulatory frameworks that integrate two distinct approaches: (1) credit-based mechanisms that link compliance records to enterprise reputation, financing eligibility, and market access; and (2) high-frequency monitoring mandates that guarantee continual oversight of workplace conditions. Evidence from multiple Organisation for Economic Co-operation and Development (OECD) countries demonstrates that simplified regulatory touchpoints and reputational incentives allow small enterprises to achieve higher compliance rates than larger corporations, thereby contesting conventional theories in regulatory studies (19–21). These findings collectively demonstrate that contemporary post-industrial contexts require a fundamental reevaluation of size-based compliance assumptions. This reevaluation extends beyond compliance patterns to the nature of hazards themselves.

The “Dual-Track Hazard Evolution” pattern reflects the contemporary post-industrial transitions being documented worldwide. In their systematic review, Schulte et al. (22) identified emerging hazards in future work scenarios, showing how traditional industrial hazards are declining while new risks simultaneously emerge in technology-intensive sectors. This bifurcation is supported by recent occupational health literature, which demonstrates that while conventional manufacturing exposures decrease through established control measures, novel risks in research facilities and service sectors frequently lack adequate regulatory frameworks (23, 24). These international patterns underscore the critical need for adaptive regulatory frameworks capable of simultaneously addressing both declining traditional hazards and emerging risks in post-industrial urban environments. Together, these two concepts—the Credit-Frequency Compliance Paradox and Dual-Track Hazard Evolution—provide a theoretical framework for understanding occupational health governance in post-industrial transitions. They extend traditional regulatory theory by demonstrating how contemporary hybrid governance mechanisms and sectoral transformations fundamentally alter established relationships between enterprise characteristics and compliance outcomes. However, the actual validation of these theoretical patterns in the rapidly evolving Chinese post-industrial context remains unexamined.

Despite the growing urgency, there is a deficiency of studies about occupational health adaptive strategies for swiftly expanding metropolitan populations. Additionally, research has shown that the incidence of occupational diseases and injuries among urban workers significantly exceeds the national average, demanding immediate concern for their health (25, 26). Moreover, previous studies have often overlooked temporal trends, including the COVID-19 period, as well as the interactive impacts among firm size, industry, and hazard type. In particular, investigations into nascent sectors, like research and development-intensive industries and the service sector, are still insufficient. The emergence of these new industries requires a reassessment of conventional hazard control approaches originally formulated for heavy industries. The global proliferation and swift advancement of Industry 4.0 manufacturing transformations have equipped workers with sophisticated technical tools, while simultaneously presenting new occupational health and safety hazards (27).

To address these gaps, this study conducts a comprehensive analysis of periodic occupational hazard monitoring in Tianhe District from 2020 to 2024. This work aims to quantify the Credit-Frequency Compliance Paradox using stratified monitoring data, outline Dual-Track Hazard Evolution through sector-specific hazard profiling, and offer evidence-based suggestions for occupational health governance in post-industrial urban environments. We specifically address three research questions: Does occupational health monitoring compliance adhere to the conventional pattern where larger firms exhibit higher compliance rates, or does a paradoxical reversal occur under hybrid governance frameworks? How do occupational hazard profiles change across various industry sectors and firm sizes during post-industrial transitions? What is the relationship between enterprise attributes and hazard exceedance rates for developing risks, such as noise, in high-tech and service sectors? Based on the international evidence reviewed, we propose the following hypotheses: (H1) Small and micro enterprises will display superior periodic monitoring coverage rates compared to large and medium-sized enterprises within Tianhe’s credit-based regulatory framework, contrary to traditional compliance theory; (H2) Traditional occupational hazards (dust) will demonstrate decreasing exceedance rates, while emerging hazards (noise) will exhibit increasing trends, particularly in the scientific research and technical services sectors; and (H3) Noise intensity and exceedance rates will differ significantly across enterprise sizes and industry sectors, with the highest risks emerging in previously under-monitored high-tech industries.

## 2 Materials and methods

### 2.1 Data source

We acquired data from periodic hazard monitoring reports provided by accredited occupational health service agencies for 245 enterprises via the “Guangdong Province Occupational Health Quality Control Platform,” covering the period from January 1, 2020, to December 31, 2024. We gathered data on enterprises declaring occupational hazards in Tianhe District through the Occupational Disease Hazard Program Declaring System.

### 2.2 Hazard exceedance determination and enterprise classification

Based on Occupational Exposure Limits for Hazardous Agents in the Workplace, Part 1: Chemical Hazardous Agents (GBZ 2.1—2019) (28) and Occupational Exposure Limits for Hazardous Agents in the Workplace, Part 2: Physical Agents (GBZ 2.2—2007) (29), we assessed whether each monitoring point for occupational hazards surpassed the permissible exposure limit. Enterprise size was categorized based on the Statistical Classification of Large, Medium, Small, and Micro Enterprises (30), while industry classification adhered to Industrial Classification for National Economic Activities (GB/T 4754—2017) (31). If test results for any occupational disease hazards or the concentration levels at any monitoring points of an enterprise surpass its occupational exposure limit, that enterprise will be catagarized as exceeding the standard (i.e., an exceedance enterprise). Periodic monitoring coverage rate = (number of enterprises that conducted monitoring and reported the results to the platform / number of enterprises that declared occupational hazards) × 100%. Exceedance enterprise rate = (number of enterprises with any exceedance / total number of enterprises that conducted monitoring) × 100%. Monitoring point exceedance rate = (exceeding points / total number of monitoring points) × 100%. We assessed the periodic monitoring coverage rate and exceedance enterprise rate by enterprise size, subsequently examining the exceedance status of the three major categories of occupational hazards (dust, chemical, and physical) across diverse enterprise sizes and industries.

### 2.3 Quality control

All data were acquired from the “Guangdong Province Occupational Health Quality Control Platform,” and we exclusively selected certified institutions possessing the requisite legal requirements; all their sampling equipment had been calibrated and remained within its valid certification period. Personnel involved in field sampling, laboratory testing, and data reporting at each licensed institution completed standardized training and successfully passed competency evaluations. During the data entry and summarizing process, issues such as missing data, duplicate entries, or logical mistakes are double-checked and confirmed to be rectified or removed. Noise measurements were conducted in accordance with the Measurement of Physical Agents in the Workplace Part 8: Noise (GBZ/T 189.8—2007) (32). Calibrated sound level meters were used to record equivalent continuous A-weighted sound pressure levels (L_EX,8h_ or L_EX,40h_) at representative workstations during normal production conditions. Dust sampling followed the Determination of Dust in the Air of Workplace (GBZ/T 192—2007) (33), using personal air samplers placed in workers’ breathing zones during typical work shifts. Sampling points were chosen following the Specifications of Air Sampling for Hazardous Substances Monitoring in the Workplace (GBZ 159—2004) (34), which recommends selecting representative locations that reflect workers’ actual exposure during routine production.

### 2.4 Statistical analysis

**Figure 1:**
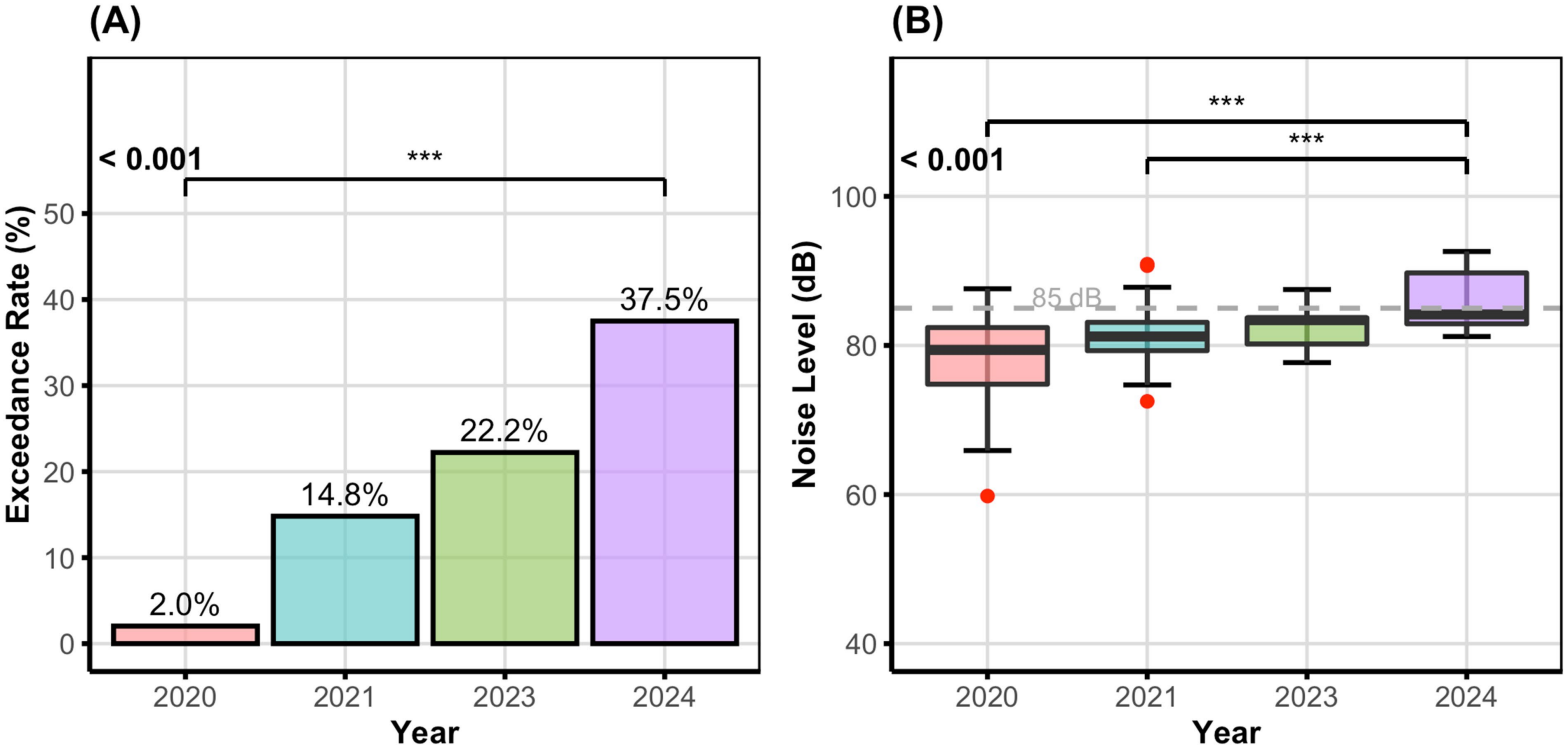
Noise hazard evolution in the scientific research and technical services industry (2020-2024). Panel **(A)** shows the rising trend in noise exceedance rates from 2.0% (2020) to 37.5% (2024). Panel **(B)** shows boxplots of noise levels across years, with the horizontal dashed line indicating the occupational exposure limit (85 dB(A)). Statistical analysis was conducted using two different trend tests: the χ² trend test for exceedance rates and the Jonckheere-Terpstra test for noise levels (both *p* < 0.001). Post-hoc pairwise comparisons were performed using Bonferroni-adjusted tests: chi-square tests for exceedance rates and Mann-Whitney U-tests for noise levels. Significance levels: *p < 0.05 **p < 0.01 ***p < 0.001. LEX,8h: Equivalent continuous A-weighted sound pressure level normalized to an 8-hour working day. LEX,40h: Equivalent continuous A-weighted sound pressure level normalized to a 40-hour working week. Data for 2022 is missing due to absence of monitoring in this sector during that year.

Data were analyzed using *SPSS 27.0 (SPSS Inc., Chicago, IL, USA)* for statistical tests and *R software (version 2024.12.1 + 563)* for data visualization. Continuous variables violating normality (assessed via Shapiro–Wilk/Kolmogorov–Smirnov tests) were summarized as median (interquartile range, IQR). Non-normally distributed silica dust concentrations were compared between industries using the Mann–Whitney U-test, while noise intensity levels (L_EX,8h_/L_EX,40h_) across enterprise sizes and industries were analyzed with the Kruskal-Wallis test, followed by post-hoc pairwise Mann–Whitney U-tests and Bonferroni correction (adjusted *α* = 0.05/m, where m denotes the number of comparisons). Line graphs generated with R visualize the temporal trends in exceedance rates for dust, chemical, and physical hazards from 2020 to 2024. Boxplots generated in R visualized temporal noise intensity for different enterprise sizes and industries. Categorical data (e.g., exceedance rates) were examined utilizing the χ^2^ trend test, Pearson χ^2^ test, or Fisher’s exact test (for small samples). For temporal analysis of the scientific research and technical services industry, the Jonckheere-Terpstra test examined linear trends in noise intensity levels from 2020 to 2024, while the χ^2^_trend test assessed variations in noise exceedance rates. Pairwise comparisons across years employed Bonferroni-adjusted chi-square tests for exceedance rates and Bonferroni-adjusted Mann–Whitney U-tests for noise levels. Boxplots combined with bar charts in Figure 1 visualized the temporal evolution of noise levels and exceedance rates in the scientific research and technical services sector. When a significant difference was found among multiple groups, pairwise comparisons were conducted with Bonferroni-adjusted *α* (α/m). The significance level was set at α = 0.05 (two-tailed). Given the inherent differences in sample sizes across enterprise categories (e.g., large enterprises were few compared with micro enterprises), statistical comparisons should be interpreted with caution. Such imbalances may affect statistical power and reduce the precision of estimates for smaller groups. Non-parametric tests were employed where appropriate to address potential violations of distributional assumptions.

Data Monitoring for the scientific research and technical services sector was unavailable for 2022 owing to COVID-19 pandemic-related interruptions in routine occupational health surveillance. Data for this period was omitted from the industry’s time trend analysis, with missing values not imputed. Trend tests (χ^2^_trend and Jonckheere-Terpstra tests) were conducted using the available data points from 2020, 2021, 2023, and 2024, yielding 101 monitoring points across the four years. The absence of data for this year may create uncertainty in the estimated rate of change and diminish statistical power for identifying temporal trends; however, the consistent upward trend evident in the other years and the achieved statistical significance indicate that the overall conclusions remain robust.

## 3 Results

Table 1 presents the periodic testing of occupational disease hazards across 245 enterprises and examines the trend in the periodic testing rate based on enterprise size. Between 2020 and 2024, 245 firms in Tianhe District, Guangzhou conducted periodic occupational hazard monitoring, out of 560 enterprises that submitted occupational hazard declarations, resulting in a periodic monitoring coverage rate of 43.75%. The rate of periodic monitoring coverage rose as enterprise size diminished, with rates of 22.22, 24.18, 47.35, and 50.60% for large, medium, small, and micro enterprises, respectively. This trend was statistically significant (χ^2^_trend = 16.987, *p* < 0.001) (see Table 1).

**Table 1:** Periodic monitoring conducted by enterprises of varied sizes in Tianhe district, Guangzhou, 2020–2024.

Enterprise size	Enterprises declared (*N*)	Enterprises monitored (*N*)	Periodic monitoring coverage rate (%)
Large	18	4	22.22
Medium	91	22	24.18
Small	283	134	47.35
Micro	168	85	50.60
Total	560	245	43.75

χ2_trend = 16.987, *p* < 0.001. Enterprise size classified according to Statistical Classification of Large, Medium, Small, and Micro Enterprises (30). Enterprises declared: Number of enterprises that declared occupational hazards. Enterprises monitored: number of enterprises that conducted monitoring and reported the results to the platform.

**Figure 2:**
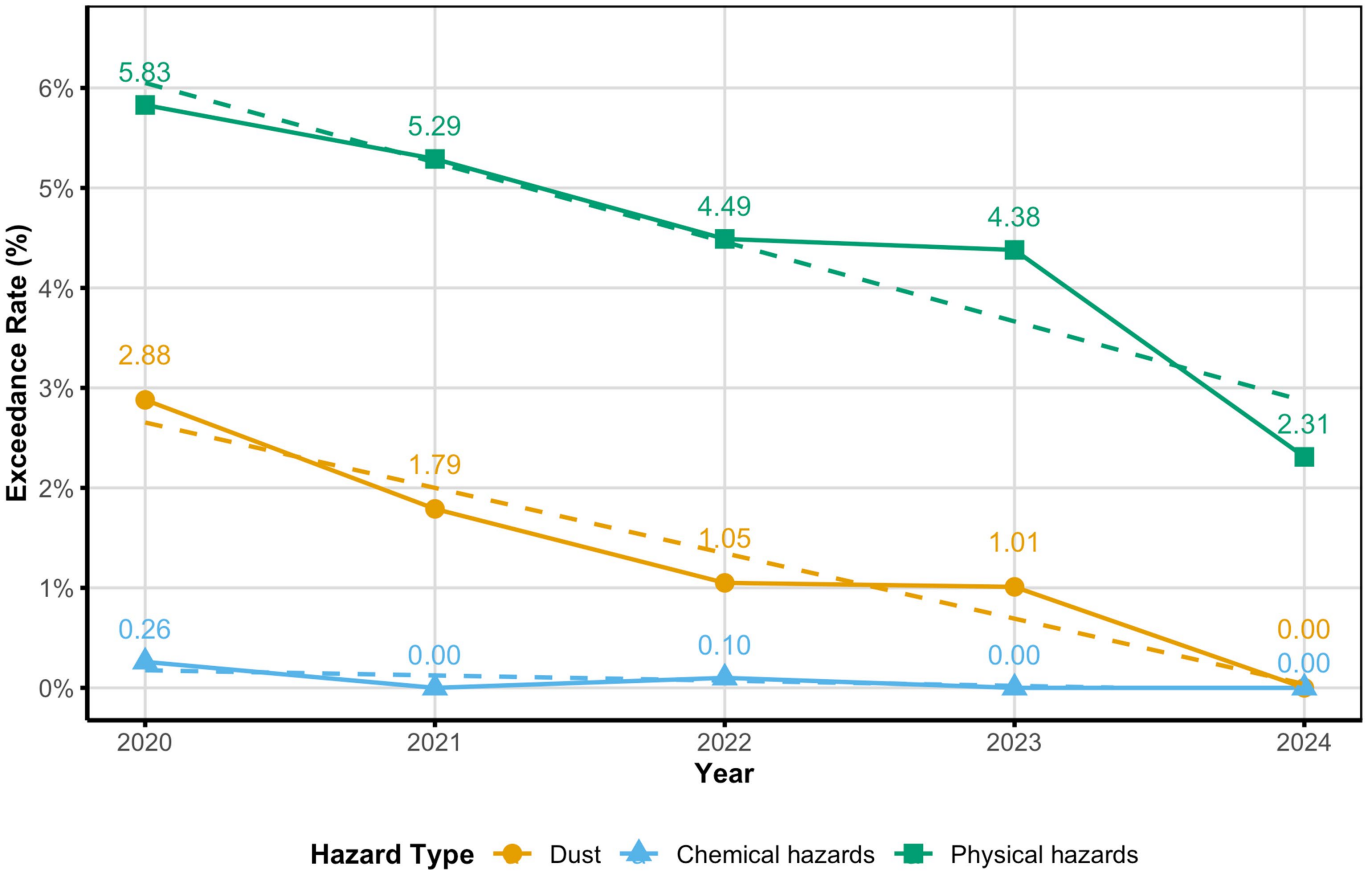
Temporal Trends in Hazard Exceedance Rates (2020–2024). Chi-squared trend analysis: χ²(Dust) = 4.965; χ²(Chemical) = 3.990; χ²(Physical) = 10.386; all *p* < 0.05. Exceedance rate refers to the percentage of monitoring points exceeding safety thresholds.

Figure 2 depicts the temporal trends in exceedance rates for dust, chemical, and physical hazards at monitoring points from 2020 to 2024. Among the 245 firms that performed periodic occupational hazard monitoring, 48 experienced exceedances, resulting in an overall exceedance enterprise rate of 19.59%. In the last five years, exceedance rates at monitoring stations for dust, chemical, and physical hazards shown a consistent year-on-year decrease, with all differences being statistically significant (*p* < 0.05). The exceedance rate for dust hazards decreased from 2.88% in 2020 to 0.00% in 2024. Similarly, chemical hazard exceedance rates drastically dropped from 0.26 to 0.00% throughout the same timeframe. The physical hazard category, predominantly characterized by noise, also witnessed a decline in exceedance rates from 5.83 to 2.31% during the study period (see Figure 2).

Over the last five years, the dust types that surpassed the permissible limits were carbon black dust, silica dust, and talc dust, with exceedance rates of 33.33% (1/3), 8.33% (7/84), and 5.56% (1/18), respectively. No statistically significant variation in exceedance was seen across these dust kinds (χ^2^ = 2.562, *p* = 0.278). Subsequent examination of the exceedance of these three dust types across various industries and enterprise sizes indicated that the exceedance rate of silica dust differed markedly by industry (χ^2^ = 13.576, *p* < 0.001). The exceedance rate for silica dust was 75.00% (6/8) in clay brick and block manufacture, compared to 5.56% (1/18) in cement products manufacturing; the overall exceedance rate in the manufacturing sector was 15.22% (7/46). The median time-weighted average concentration (C_TWA_) of silica dust exhibited substantial variation across industries (U = 4.000, *p* < 0.001). The median silica dust C_TWA_ was greatest in clay brick and block manufacture (0.76 mg/m^3^) and least in cement products manufacturing (0.19 mg/m^3^). No statistically significant variation in dust exceedance was seen across enterprise sizes (χ^2^ = 1.962, *p* = 0.580).

Within the past five years, the chemical factors that exceeded permissible limits were 1,2-dichloroethane, ozone, and xylene, with exceedance rates of 5.00% (1/20), 0.97% (1/103), and 0.21% (1/484), respectively. The exceedance rate of chemical risks varied markedly among industries (χ^2^ = 78.753, *p* < 0.001). The exceedance rate was 12.50% (1/8) in other unclassified service industries, whereas it was 0.10% (2/1919) in vehicle repair and maintenance. All exceeded chemical factors are found in the “residents’ services, repair, and other services” sector (100%), with an exceedance rate of 0.15% (3/2019). No statistically significant variation in chemical hazard exceedance was seen across firms of varying sizes (χ^2^ = 0.749, *p* = 0.862).

Table 2 shows the exceedance rates among different physical hazards. In the last five years, the physical hazards that exceeded permissible limits included noise, high temperature, and ultraviolet radiation (welding arc), with exceedance rates of 5.00% (119/2380), 4.02% (7/174), and 0.67% (1/149), respectively. The disparities in exceedance rates among these physical parameters were statistically significant (χ^2^ = 6.058, *p* = 0.048). A two-by-two comparison using the Bonferroni method revealed a statistically significant difference in the rate of exceedance between noise and ultraviolet radiation (welding arc) (χ^2^ = 5.814, *p* = 0.016) (see Table 2).

**Table 2:** Exceedance rates among different physical hazard monitoring points, 2020–2024.

Physical hazard type	Monitoring points (*N*)	Exceeding points (*N*)	Exceedance rate (%)
Noise	2,380	119	5.00^a^
High temperature	174	7	4.02^a,b^
Ultraviolet radiation (welding arc)	149	1	0.67^b^
Total	2,703	127	4.70

Overall difference significant by χ2 test (χ2 = 6.058, *p* < 0.05). Post hoc pairwise χ2 test showed significant difference between noise and ultraviolet radiation (χ2 = 5.814, *p* < 0.05).

We further conducted a comprehensive analysis of noise exceedance rates across various firm sizes and industries with noise exceedance. Table 3 illustrates the findings for noise monitoring exceedance rates categorized by enterprise size. The noise exceedance rate decreased as enterprise size decreased (from 12.80% in large firms to 3.43, 5.29, and 1.39% in medium, small, and micro firms, respectively), and this trend was statistically significant (χ^2^_trend = 13.879, *p* < 0.001). The data indicates that larger enterprises are more prone to exceed permissible noise levels than smaller firms. We also analyzed the exceedance rates of noise across various industries. The scientific research and technical services sector displayed the highest exceedance rate at 12.87% (13/101), followed by the manufacturing sector, which showed an exceedance rate of 7.89% (88/1115). The disparities in noise exceedance rates across industries were statistically significant (χ^2^ = 37.836, *p* < 0.001). These findings highlight the growing danger of noise exposure in specific sectors, especially in high-tech research settings and manufacturing factories (see Table 3).

**Table 3:** Exceedance rates of noise monitoring points by enterprise size and industry, Tianhe district, Guangzhou, 2020–2024.

Category	Monitoring points (*N*)	Exceeding points (*N*)	Exceedance rate (%)
Enterprise size			
Large	164	21	12.80^a^
Medium	437	15	3.43^b,c^
Small	1,492	79	5.29^c^
Micro	287	4	1.39^b^
Subtotal	2,380	119	5.00
Enterprise industry			
Electricity, heat, gas, and water production and supply	269	4	1.49^a^
Residents’ services, repairs, and other services	552	14	2.54^a^
Scientific research and technical services	101	13	12.87^b^
Manufacturing	1,115	88	7.89^b^
Subtotal	2037	119	5.84

Different superscripts (a, b, c) indicate statistically significant differences in exceedance rates between groups within each category (i.e., Enterprise Size and Enterprise Industry) based on Bonferroni-adjusted chi-square tests, *p* < 0.05. χ2_trend for exceedance rates by enterprise size = 13.879, *p* < 0.001. χ2_trend for exceedance rates by enterprise industry = 37.836, *p* < 0.001. Exceedance rate defined as (number of exceeding monitoring points/total monitoring points) × 100%.

**Figure 3:**
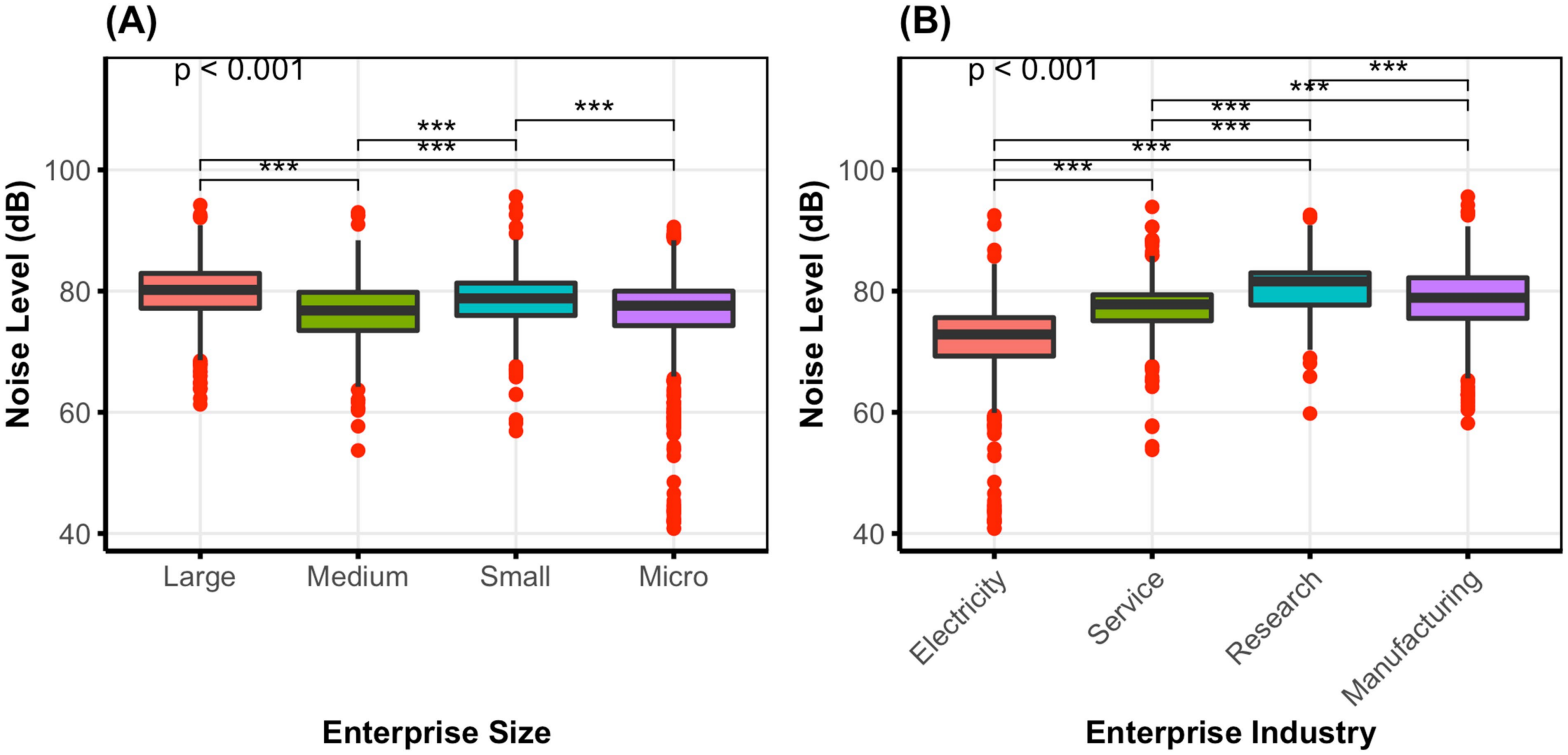
Noise exposure disparities across enterprise sizes and industries (2020–2024). Boxplots **(A)** and **(B)** show median LEX,8h/LEX,40h with IQRs, whiskers, outliers, and Bonferroni-adjusted pairwise significance. Statistical analysis was conducted using the Kruskal-Wallis test (H = 55.140, *p* < 0.001 for enterprise sizes; H = 254.964, *p* < 0.001 for enterprise industries), with post-hoc pairwise comparisons using the Bonferroni method. Bonferroni-adjusted Mann–Whitney U-tests significance levels: *p < 0.05 **p < 0.01 ***p < 0.001. LEX,8h: Equivalent continuous A-weighted sound pressure level normalized to an 8-hour working day. LEX,40h: Equivalent continuous A-weighted sound pressure level normalized to a 40-hour working week. Abbreviations: Electricity: Electricity, heat, gas, and water production and supply; Service: Residents’ services, repairs, and other services; Research: Scientific research and technical services.

Afterwards, Figure 3 depicts the median workplace noise levels (L_EX,8h_/L_EX,40h_) over various enterprise sizes and industries. The data, presented as boxplots, reveal substantial variation in median noise levels across both enterprise sizes (H = 55.140, *p* < 0.001) and industries (H = 254.964, *p* < 0.001). The median workplace noise levels across all enterprise sizes stayed below 85 dB(A), but with substantial variations within the size categories. The scientific research and technical services sector found the greatest median noise level at 81.60 dB(A), while the electricity, heat, gas, and water production and supply sector had the lowest median noise level at 72.85 dB(A). Figure 3 further amplifies these variations by visually illustrating the discrepancies in noise intensity levels (see Figure 3).

The temporal analysis of noise hazards in the scientific research and technical services industry (Table 4; Figure 1) revealed an alarming rise in both exceedance rates and noise intensity levels from 2020 to 2024. The noise exceedance rate in this sector rose considerably from 2.04% in 2020 to 37.50% in 2024 (χ^2^_trend = 14.318, *p* < 0.001). Simultaneously, median noise intensity levels (L_EX,8h_/L_EX,40h_) increased from 79.40 dB(A) in 2020 to 84.15 dB(A) in 2024, bringing 2024 levels greatly above those observed in both 2020 and 2021 (Jonckheere-Terpstra test = 2532.000, *p* < 0.001). This concerning trajectory emphasizes the scientific research and technical services sector as the most evident illustration of the growing risk pattern discovered in our study. No data was available for 2022 owing to the absence of monitoring in this area during that year.

**Table 4:** Exceedance rates and median noise exposure levels (L_EX,8h_/L_EX,40h_) among Scientific research and technical services industry, 2020–2024.

Year	Monitoring points (*N*)	Exceeding points (*N*)	Exceedance rate (%)	L_EX,8h_/L_EX,40h_ dB(A) [M(Q1 ~ Q3)]
2020	49	1	2.04^a^	79.40 (74.80 ~ 82.40)
2021	27	4	14.81^a,b^	81.20 (79.20 ~ 83.50)
2023	9	2	22.22^a,b^	83.40 (79.40 ~ 85.45)
2024	16	6	37.50^b^	84.15 (82.90 ~ 89.98)***
Total	101	13	12.87	81.60 (77.65, 83.10)

a, b: Significant differences in exceedance rates between years (Bonferroni-adjusted chi-square tests, *p* < 0.05). *, **, ***: Significant differences in noise levels between years (Bonferroni-adjusted Mann–Whitney U-tests, *p < 0.05, **p < 0.01, ***p < 0.001). *** for 2024 indicates significant differences compared to both 2020 and 2021. L_EX,8h_: Equivalent continuous A-weighted sound pressure level normalized to an 8-h working day. L_EX,40h_: Equivalent continuous A-weighted sound pressure level normalized to a 40-h working week. Trend analysis showed significant increasing trends for both exceedance rate (χ^2^_trend = 14.318, *p* < 0.001) and noise levels (Jonckheere-Terpstra test = 2532.000, *p* < 0.001). 2022-year data missing due to the absence of noise exposure data for the Scientific research and technical services industry in the original monitoring data.

## 4 Discussion

### 4.1 The credit-frequency compliance paradox: local evidence, global implications

The inverse size-compliance relationship in Tianhe District—small and micro enterprises attaining 47.35–50.60% compared to large and medium enterprises’ 22.22–24.18%—stands in stark contrast to trends observed in other Chinese regions (χ^2^_trend = 16.987, *p* < 0.001). Panyu District in Guangzhou (35) and Kaifeng City (36) exhibit typical gradients where compliance escalates with firm size. Comparable international evidence supports this pattern. For instance, Scruggs et al. (37) found that company size is a key determinant in understanding and complying with the European Union (EU)’s Registration, Evaluation, Authorization, and Restriction of Chemicals (REACH) regulation, with larger firms demonstrating greater compliance potential. The regional variation within China suggests that Tianhe’s hybrid governance mechanism, combining credit-based penalties with high-frequency monitoring, triggers unique compliance dynamics absent in traditional regulatory approaches.

In practice, Tianhe District operationalizes this hybrid governance model through two core mechanisms. The credit component functions within Guangzhou’s enterprise credit management framework, in which occupational health compliance records are systematically documented and disclosed; enterprises with regulatory violations face credit score penalties that affect their eligibility for government procurement contracts, preferential financing, and market reputation—establishing concrete economic incentives for compliance beyond traditional punitive measures, consistent with evolving regulatory strategies in China’s occupational health governance (38). The frequency component is implemented via the Guangdong Province Occupational Health Quality Control Platform, which mandates regular hazard assessments at fixed intervals and supports real-time monitoring of corporate compliance. Non-compliant firms face escalating administrative penalties and public disclosure, resulting in sustained regulatory pressure that differs markedly from the irregular enforcement typical of traditional approaches.

Several factors may account for this unexpected pattern. First, Tianhe’s market-oriented third-party testing services have diminished compliance costs for SMEs, alongside specific governmental support measures (39, 40). Second, small and micro enterprises in Tianhe predominately function within high-risk sectors (e.g., manufacturing, services), generating more robust compliance incentives compared to regions with different industrial compositions. Third, the inverted-U relationship between firm size and compliance costs identified by Trebbi et al. (14) suggests small and micro firms benefit from regulatory tiering while medium enterprises face maximum burden and large enterprises experience implementation delays from complex management structures.

### 4.2 Dual-track hazard evolution: traditional declines, emerging risks

The divergent hazard trajectories—decreasing traditional exposures alongside the emergence of new risks—align with Schulte et al.’s (22) systematic review of prospective work scenarios. Nonetheless, Tianhe’s pattern displays distinctive characteristics. Despite national legislation and enhanced dust suppression techniques improving compliance in other sectors (41, 42), silica dust concentrations in clay brick and block manufacturing persistently exceed limits by 75.00%, illustrating that certain industrial sub-sectors continue to pose challenges. This contrasts sharply with the successful dust control achieved in traditional mining, indicating regulatory blind spots within specific industrial processes. The emergence of chemical hazards in unclassified service industries, with a 12.50% exceedance rate, contradicts the traditional emphasis on manufacturing and construction as principal sources of chemical exposure, where studies have reported prevalence rates of 60–76% (43). This implies that post-industrial transitions may shift detrimental chemical exposures to neglected workplaces.

Most striking, however, is the trajectory of noise hazards. The scientific research and technical services sector showed a 12.87% exceedance [median 81.60 dB(A)] in contrast to manufacturing’s 7.89%, challenging traditional risk hierarchy (44). This discovery transcends Tianhe—The Occupational Safety and Health Administration (OSHA) indicates that certain large analytical instruments, tissue homogenizers, stirring motors, and associated mechanical systems in laboratories can produce noise levels exceeding 85 dB(A), and in extreme instances, approaching 90 dB due to the cumulative effects of multiple devices and spatial reflections (45). The temporal increase in the scientific research and technical services industry from 2.04% (2020) to 37.50% (2024) exceedance, with median levels attaining 84.15 dB(A), signifies an 18-fold escalation during a timeframe when other threats diminished. This tendency, statistically significant (χ^2^_trend = 14.318, *p* < 0.001; Jonckheere-Terpstra test = 2532.000, *p* < 0.001), suggests systematic rather than incidental exposure growth across knowledge-intensive sectors previously deemed low-risk. Recent occupational epidemiological research reinforces this concern: scientific research and technical services now present a heightened risk for hearing impairment, comparable to that of manufacturing, indicating that these settings are no longer marginal in noise exposure studies (46).

### 4.3 Regional context and economic implications

Tianhe’s position as Guangzhou’s leading GDP-contributing area exacerbates occupational health issues. The established association between enterprise-level noise-induced hearing loss (NIHL) incidence and regional GDP (*r*_s_ = 0.879, *p* < 0.01) (4) indicates a latent risk, although the absence of reported cases from 2020 to 2024. The current monitoring coverage stands at 43.75%, above provincial (5.9%) and municipal (4.4%) averages (2), however it reveals significant surveillance deficiencies, especially in burgeoning high-tech sectors.

The median noise levels in the scientific research and technical services sector are approaching the 85 dB(A) regulatory threshold over years. Comparable tendencies observed in other innovation-oriented centers. Alqudah et al. identified that 16.67% of dental laboratory workers exhibited hearing impairment in urban technical facilities (47). Recent campus measurements in Ecuador indicated peak noise levels surpassing 90 dB(A), underscoring dangers in university environments that serve as knowledge-intensive centers (48). In Europe, reviews of Industry 4.0 similarly underscore noise as an emerging risk in research and innovation settings (27). Without intervention, Tianhe’s Research and Development (R&D) industry threatens to emerge as the principal contributor to occupational hearing loss, undermining advancements made in traditional manufacturing via established control mechanisms.

### 4.4 Urban occupational health governance

The intersection of compliance inversion and hazard bifurcation undermines traditional regulatory assumptions. Tianhe’s hybrid governance success exemplifies alternatives to uniform regulatory frameworks that apply uniform standards regardless of firm attributes. Implementation necessitates recognition of established constraints: resource limitations (13), insufficient regulatory awareness in smaller firms (12), and the inverted-U burden distribution where medium-sized enterprises incur the highest compliance costs (14).

The effectiveness of Tianhe’s credit-frequency mechanisms may depend on specific institutional and economic conditions. The high concentration of SMEs in high-risk sectors and governmental support strategies may not exist elsewhere. Nevertheless, these adaptive regulatory principles- customized to local industrial ecosystems- may still offer valuable insights for other post-industrial settings. Their broader applicability, however, remains an open question and will require further empirical examination.

## 5 Conclusion

### 5.1 Theoretical contributions

This research develops two innovative conceptual frameworks for urban occupational health governance. The “Credit-Frequency Compliance Paradox” quantifies the conditions in which hybrid governance measures effectively reverse typical size-compliance dynamics. The “Dual-Track Hazard Evolution” interpretive framework proposed by the authors outlines the bifurcated nature of occupational risk transitions throughout post-industrial economic transformations.

These frameworks provide initial quantitative tools for measuring phenomena that have largely been discussed only at a conceptual level in the existing literature. Although they were developed within Tianhe’s specific institutional context, the proposed methods may still help guide future analyses of compliance patterns and risk dynamics in comparable urban settings, pending validation in a broader range of environments.

### 5.2 Evidence-based recommendations

Based on our empirical findings and contemporary international evidence, we advocate for targeted interventions.

#### 5.2.1 Adaptive regulatory approach

Recent systematic reviews demonstrate that specific occupational health and safety (OHS) policy mechanisms can significantly decrease workplace injury rates and improve regulatory compliance (49, 50). An evidence and gap map examining OHS regulatory interventions across OECD countries found that inspection efforts and enforcement tactics exhibit measurable effectiveness, despite implementation varies considerably by regional context (18).

By describing the historical evolution of OSH regulations and “inspectorates” in countries such as France, Germany, the Netherlands, and the United States, Blanc et al.’s research reveals how regulatory interventions, rulemaking, and inspection activities dynamically adapt to the emergence of new technologies and industries (51). This adaptive approach emphasizes that regulatory efficacy is determined not by static frameworks, but by dynamic adaptation to local industry evolution and cultural circumstances.

#### 5.2.2 Targeted hazard mitigation

For persistent hazards identified—75.00% silica dust exceedance in clay brick and block manufacturing, 12.50% chemical risks in unclassified service industry, and 18-fold noise escalation in the scientific research and technical services sector—we recommend evidence-based control hierarchies. A systematic review of 146 workplace intervention studies found engineering controls constituted the most common intervention type (43% of studies), with consistent evidence of effectiveness when properly implemented (52). The efficiency of local exhaust ventilation (LEV) for tucking point grinders and a portable abrasive cutter in a bricklayers’ training center was evaluated using a randomized block design (52). As compared to the control, the LEV device dramatically decreased the mean silica dust concentrations in the workers’ breathing zone by up to 96% (52). Additionally, learn from the U.S. Environmental Protection Agency (EPA) and the European Union, which have implemented the Registration, Evaluation, Authorization, and Restriction of Chemicals (REACH) rule, creating a “white list” system for chemicals in newly unclassified services (53). Adopt strategies to eliminate or limit the utilization of industrial solvents that present serious hazards to human health, and advocate for the adoption of safer industrial solvents or adhesives (53). Moreover, a layer approach combing source control (quieter machines), path control (noise barriers), and receiver protection (soundproof windows, earmuffs) can provide comprehensive noise management (54).

Notably, Bluetooth-enabled Pyramex VentyreGear AmpBT earmuffs, one of the most recommended earmuffs, automatically activate when noise levels exceed 85 decibels (dB), effectively reducing noise by 26 dB to below the acceptable threshold (54).

#### 5.2.3 Technological integration

Internet of Things (IoT) and artificial intelligence can enable continuously monitor working conditions in real time. A safer working environment is promoted by this ongoing monitoring capabilities, which dramatically lowers workplace accidents and occupational illnesses (55). As demonstrated by studies, during a two-year period, organizations that implemented such technologies observed a 25% decrease in occupational injuries (55).

### 5.3 Limitations and future directions

Although this research offers actionable insights, it is constrained by potential sampling bias toward registered enterprises, the limited availability of certain hazard measurements, and pandemic-related data gaps. The 2022 absent data for the scientific research and technical services sector might potentially impact trend analysis for that industry. Our findings also lack validation from independent external datasets, constraining the generalizability of our proposed frameworks. This analysis only included registered businesses with formal monitoring programs; thus, the actual levels of occupational hazard exposure may be underestimated. This is because unregistered or non-compliant enterprises potentially facing higher risk exposures were not included. Additionally, as outlined in Appendix A, most enterprises included in the monitoring program varied from year to year. Only 4.49% of companies were monitored continuously throughout the entire study period, indicating a predominantly cross-sectional rather than a longitudinal cohort design.

Furthermore, when comparing large and medium-sized enterprises with small and micro firms, inherent differences in sample sizes across groups may affect statistical power. Although trend tests and stratified analyses were applied to mitigate this issue, the unequal distribution of enterprises across size categories remains an important limitation when interpreting these results. Future research should validate the proposed frameworks across diverse geographic and economic contexts, investigate specific noise sources in research facilities, and examine the scalability of hybrid governance mechanisms beyond Tianhe’s unique conditions.

## Data Availability

The raw data supporting the conclusions of this article will be made available by the authors, without undue reservation.

## Appendix A

### Enterprise sample characteristics and tracking pattern

1. Number and size of enterprises

Out of 560 enterprises that declared occupational hazards in Tianhe District during 2020 to 2024, 245 enterprises (43.75%) performed periodic monitoring and submitted data for analysis.

The 245 monitored enterprises had the following size distribution:

- Large enterprises: 4 (1.63% of monitored enterprises)
- Medium enterprises: 22 (8.98% of monitored enterprises)
- Small enterprises: 134 (54.69% of monitored enterprises)
- Micro enterprises: 85 (34.69% of monitored enterprises)

1. Enterprise tracking pattern

We analyzed monitoring frequency of the 245 enterprises across the five-year period to determine whether enterprises were consistently tracked:

- Monitored all 5 years (2020–2024): 11 enterprises (4.49%)
- Monitored in 4 different years: 24 enterprises (9.80%)
- Monitored in 3 different years: 30 enterprises (12.24%)
- Monitored in 2 different years: 40 enterprises (16.33%)
- Monitored only once: 140 enterprises (57.14%)

Our analysis shows the enterprises were largely NOT the same from year to year. Only 11 out of 245 enterprises (4.49%) maintained continuous monitoring throughout the study period. The majority (57.14%) appeared in our dataset only once during the five years, indicating substantial turnover in the monitored population.

This cross-sectional sampling pattern, rather than a longitudinal cohort design, illustrates the dynamic nature of Tianhe’s business environment and the fluctuating compliance patterns. While this represents a limitation for enterprise-level longitudinal analysis, it does not affect the validity of the district-wide hazard trends reported in the main manuscript.

Data Source: Guangdong Province Occupational Health Quality Control Platform (2020–2024).
